# The Midland Counties' Home for Incurables

**Published:** 1899-07-15

**Authors:** 


### THE MIDLAND COUNTIES' HOME FOR INCURABLES.

At the Midland Counties Home for Incurables a new wing for male patients, and domestic quarters, have just been completed and opened as a Jubilee commemoration. The block consists of three storeys, on each floor is one large and three small wards, and the usual offices, the whole accommodating thirty-three patients. The new domestic quarters accommodate twenty-three persons, the whole additional premises being carried out at a cost of about £6,800. The money has been contributed throughout the Midland Counties, Birmingham especially having contributed largely. Miss Izod of that city gave £2,000, and is furnishing one of the large wards. It is proposed to erect an ornamental water tower, as without some such arrangement an adequate supply of water in case of fire is not obtainable. Already £100 has been promised towards this object.

